# Stimulating seedling growth in early stages of secondary forest succession: a modeling approach to guide tree liberation

**DOI:** 10.3389/fpls.2014.00345

**Published:** 2014-07-18

**Authors:** Marijke van Kuijk, Niels P. R. Anten, Roelof J. Oomen, Feike Schieving

**Affiliations:** ^1^Department of Biology, Institute of Environmental Biology, Utrecht UniversityUtrecht, Netherlands; ^2^Department of Crop and Weed Ecology, Centre for Crop Systems Analysis, Wageningen UniversityWageningen, Netherlands; ^3^Crop Science Group, Institute of Crop Science and Resource Conservation, University of BonnBonn, Germany

**Keywords:** forest restoration, gap creation, photosynthesis model, light competition, Vietnam

## Abstract

Excessive growth of non-woody plants and shrubs on degraded lands can strongly hamper tree growth and thus secondary forest succession. A common method to accelerate succession, called liberation, involves opening up the vegetation canopy around young target trees. This can increase growth of target trees by reducing competition for light with neighboring plants. However, liberation has not always had the desired effect, likely due to differences in light requirement between tree species. Here we present a 3D-model, which calculates photosynthetic rate of individual trees in a vegetation stand. It enables us to examine how stature, crown structure, and physiological traits of target trees and characteristics of the surrounding vegetation together determine effects of light on tree growth. The model was applied to a liberation experiment conducted with three pioneer species in a young secondary forest in Vietnam. Species responded differently to the treatment depending on their height, crown structure and their shade-tolerance level. Model simulations revealed practical thresholds over which the tree growth response is heavily influenced by the height and density of surrounding vegetation and gap radius. There were strong correlations between calculated photosynthetic rates and observed growth: the model was well able to predict growth of trees in young forests and the effects of liberation there upon. Thus, our model serves as a useful tool to analyze light competition between young trees and surrounding vegetation and may help assess the potential effect of tree liberation.

## Introduction

Disturbed or degraded primary forests can recover to a certain extent through secondary succession, which is in essence a continuous replacement of tree species. Native and exotic species that grow after disturbance affect natural forest recovery in different ways (Ortega-Pieck et al., 2011).

The presence of (remnant) native trees and grasses often create a favorable climate for other (pioneer) species to establish and recruit (Carpenter et al., 2004; Muñiz-Castro et al., 2006; Zahawi and Augspurger, 2006; Miao et al., 2013). Once pioneer species have established they can replace the herbaceous layer and create microclimatic conditions in which a more diverse community of later successional species can regenerate (Finegan, 1996; Peña-Claros, 2003; Guevara et al., 2005; Hughes et al., 2012).

However, natural forest recovery after degradation is often slow or stagnates due to excessive growth of exotic shrubs, lianas or grasses (Guariguata and Dupuy, 1997; DeWalt, 2003; Hooper et al., 2004; Davies and Semui, 2006; Esquivel et al., 2008). These species affect the establishment of woody species through competition for a variety of resources including light, water and nutrients (Holl et al., 2000; Parrotta et al., 2002; Hooper et al., 2005; Hoffmann and Haridasan, 2008; Ortega-Pieck et al., 2011). In wet tropical forests especially competition for light seems to play a predominant role in determining the course of succession (Gilbert et al., 2001; King et al., 2005; Selaya and Anten, 2010).

In recent years there has been increased attention for restoration ecology and in particular for management options to accelerate recovery of forests (Parrotta et al., 1997; Holl and Kapelle, 1999; Chazdon, 2013). One often used method to increase (native) tree growth and thus facilitate more rapid succession involves removing the aboveground parts of the vegetation surrounding target trees (Chapman and Chapman, 1999; Fuhr et al., 2001; Dolanc et al., 2003; Duncan and Chapman, 2003). This method, called liberation, can stimulate growth of these individual trees by reducing competition with neighboring plants, especially competition for light. However, liberation has not always had the desired effect (Collet et al., 1998; De Graaf et al., 1999; Otsamo, 2000; Chapman et al., 2002).

Liberation increases light levels around target trees. Studies have shown that light requirements differ among species (Ramos and del Amo, 1992; Montagnini et al., 1997; Dupuy and Chazdon, 2006), even those that are closely related (Korpelainen et al., 1995), and can change with tree age (Davidson et al., 2002). Thus, the effects of increasing light availability through liberation may differ depending on the species-specific physiological traits of the tree being liberated. A better understanding of the interaction between the target species and the surrounding vegetation is needed in order to improve the success of attempts to restore tropical forests (Hardwick et al., 2004). Doing this experimentally however, requires a lot of time, space and money and the outcome is usually restricted to a specific set of species in a specific setting and a limited amount of experimental conditions (for instance gap size) that are created (see Paquette et al., 2006 and references therein).

In this study we present a three-dimensional model (see Supplementary Material) to complement experimental research, which enables us to examine how stature, crown structure, and physiological traits of the target trees and the density and height of the surrounding vegetation in concert determine the whole-plant photosynthesis of target trees. Height, crown dimensions, leaf area, leaf angle distribution, and leaf physiological characteristics of target trees can be varied. The characteristics of the surrounding vegetation (Leaf Area Index, leaf angle distribution, height) can also explicitly be specified and the effects of various types of management practices (e.g., gap creation) can be simulated. This model is used as a first approach to determine the responses of different species to varying levels of release from light competition through liberation. As noted above in moist tropical forests light competition is important in driving early secondary succession and a primary effect of tree liberation is release from this competition.

The model was applied to a dataset from a young natural secondary degraded forest in Vietnam. In South East Asia species of the shrub *Melastoma* grow excessively on degraded lands (Davies and Semui, 2006) and are known to inhibit succession (DeWalt, 2003). Also, large areas of (secondary) forests are converted into grasslands dominated by *Imperata cylindrica* (Werger, 1983; Otsamo et al., 1997), which cause severe competition with emerging trees. Seedlings of pioneer tree species that recruited after a slash and burn treatment were monitored over time (see Van Kuijk et al., 2008). In a 1.5 years old stand half of the studied individuals were liberated of surrounding vegetation. We used model calculations to determine the effect of liberation of individual trees of three woody species in terms of light capture and photosynthesis. The validity of the model was critically tested by comparing predicted tree photosynthetic rates for a given point in time to subsequent growth rates, an approach also used in other studies on light competition (Hikosaka et al., 1999). We also simulated the effects of vegetation LAI and height and gap radius in vegetation removal events, and we predicted the effect of liberating target trees in stands of different successional status.

## Methods

### Study area

The study site is located in the buffer zone of Bach Ma National Park, Thua Thien Hue Province, in central Vietnam (16°10′N 107°50′E). Bach Ma National Park and its buffer zone were established in 1991 with a total area of 43,331 ha. It is the core of the last remaining contiguous forest belt in Vietnam, stretching from the South China Sea to the border with Laos. The area experiences high rainfall, especially from November until February (up to 8000 mm per year). There is no distinct dry season and the vegetation is evergreen (Tran and Ziegler, 2001). After chemical destruction of forests in the war, the study site was used for monoculture plantations of *Acacia mangium* for several decades. Part of the site was left fallow in 1999.

### Study species

In November 2004 we applied a slash and burn treatment in a 5-year old forest (the part that was left fallow in 1999) (see Van Kuijk et al., 2008 for details). The woody species that recruited afterwards were monitored over time. For this study the three most abundant recruiting tree species were selected: *Mallotus microcarpus*, *Mallotus paniculatus* and *Macaranga denticulata* (all Euphorbiaceae). Species of *Mallotus* and *Macaranga* are known to regenerate on deforested or degraded lands (Slik et al., 2003; Lee et al., 2005; Toma et al., 2005) and are characteristic of secondary forests in South-East Asia (Steenis, 1965; Primack and Lee, 1991). The role of these pioneer tree species in succession is important as they have the ability to overgrow grass and shrub species that may strongly hamper succession (see also Finegan, 1984, 1996).

### Measurements

Measurements were performed in April 2006, 1.5 years after the slash and burn treatment (1.5 y/o stand hereafter). Crown allometry of the individual target trees (20 individuals for *Mallotus microcarpus*, 31 for each of *Mallotus paniculatus* and *Macaranga denticulata*) was determined by measuring crown dimensions in four wind directions and from the bottom to the top of the crown. As part of another experiment on the same individuals, biomass allocation, photosynthetic characteristics and nitrogen contents, and data on the vegetation in which they grew, were measured (see Van Kuijk et al., 2008 for detailed methods). A summary of those methods is provided below.

After initial measurements, half of the individuals were selected randomly distributed over the study area to be removed of surrounding vegetation (“liberated plants”). All vegetation (shrubs, grasses, lianas etc.) was removed in a radius of 0.5 m around the stem of the selected individuals, from soil level until the top of the surrounding vegetation, so that their crowns did not interact with the surrounding vegetation. Regrowing vegetation was removed monthly. The vegetation around the other half of the individuals was left intact (“control plants”). After 174 days biomass allocation of all individuals was determined non-destructively (they were part of another ongoing study) so that growth of control and liberated trees could be calculated (for methods and calculations on non-destructive measurements see also Van Kuijk et al., 2008).

#### Biomass allocation

We measured the following parameters on all study trees: height, leaf angles, length, and diameter of stem, branches and petioles and length and width of leaves. To obtain allometric relations between dimensions and biomass of above ground plant parts, 20 individuals per species were harvested in the same height range as the studied individuals. The same dimensions were measured and dry weight of stem, braches, petioles, and leaves was determined. Dry weights and estimates based on dimensions were correlated and the function that best described dry weight (*r*^2^ varied from 0.92 to 0.99; data not shown) (but see Van Kuijk et al., 2008) was used to calculate dry weight of studied trees. Leaf area was measured with a digital photograph (SigmaScan Pro 5.0).

#### Photosynthetic characteristics

In March 2005, photosynthesis measurements were done using an open gas exchange system (CIRAS 2, PP systems, Hitchin, UK) equipped with a LED light source. Up to 28 leaves of varying age (young, medium and old: related to position on the branch) were selected on different individuals (max. three leaves per individual) that were growing outside the plots. Photosynthetic rates were measured early in the morning when stomata were open. Maximum photosynthetic rates were measured at Photosynthetic Active Radiation (PAR) values of 1200–1500 μmol m^−2^s^−1^. In order to determine dark respiration and quantum yield we varied light from 80 to 0 μmol m^−2^s^−1^ PAR in steps of 10–20 μmol m^−2^s^−1^. The CO_2_ concentration in the chamber was maintained at 370 ppm throughout all measurements.

#### Nitrogen content

Calculation of the distribution of light saturated photosynthetic rates in the tree crown was done as a function of the nitrogen (N) distribution (Hirose and Werger, 1987). *N* content was only measured in the most illuminated leaves in the top of the crown (*N_o_*) by drying sampled leaves in an oven for 72 h at 70°C. Nitrogen content of the leaves was analyzed with a continuous flow analyzer (SKALAR, Breda, the Netherlands) following the Kjeldahl method. We calculated the N distribution in the crown (*N*_area_) using the equation proposed by Anten (1997): *N*_area_ = *N_o_*(*I*/*I*_o_)^0.4^ (see Equation S11 in Supplementary Material) with *I*/*I*_o_ the relative light intensity. This equation shows that the *N* distribution scales with the light distribution by a power 0.4. It was shown to give good predictions of *N* distribution in stands of a wide variety of species (Anten, 1997).

#### Vegetation data

We established 1 m^2^ plots in the study area, each containing an individual tree in the center, in which light and LAI were measured. All measurements were done under a uniform overcast sky. Photosynthetic Photon Flux Density (PPFD, 400–700 nm) was measured in the center of each quadrant of each plot, summing up to four light profiles per study tree. These were averaged per plot. Light was measured at ground level, 0.25, 0.5, 0.75 and 1 m using spherical light quantum sensors and meters (LI-250, LiCor). Field testing revealed that light levels higher up in the canopy could be accurately calculated from these values. Simultaneously light measurements were done above the vegetation canopy. The Leaf Area Index (LAI) was measured four times in each plot at ground level from every corner of the (sub)plot facing the center (LAI-2000 Plant Canopy Analyzer, LiCor, NE, USA). Vertical leaf area distribution was determined by counting the number and recording the height of leaves touched by a telescopic rod when moved up through the vegetation. This was done in the center of each quadrant of a plot.

### Model calculations and simulations

The model developed here (PHOLIAGE-model) calculates daily canopy photosynthesis of individual 3D trees in a 3D vegetation stand. It was designed to simulate how the structure of the surrounding vegetation and the pattern in which it can be removed through liberation interact with the physiological and morphological traits of trees in determining whole-tree photosynthetic carbon gain. A detailed description of the model is provided in the Supplementary Material. Here we only describe the basic structure of the model and the simulations that were run.

**Figure 1:**
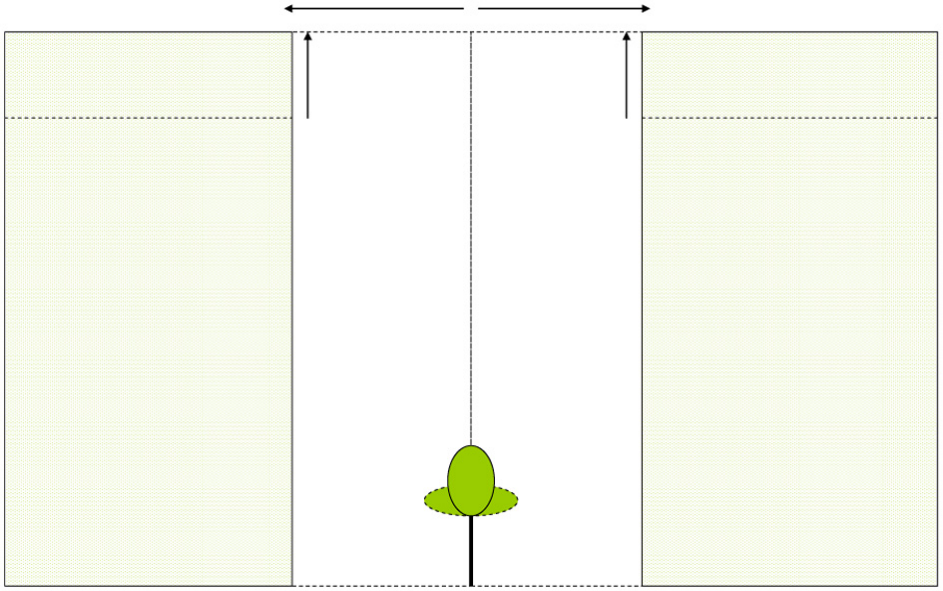
**Schematic presentation of a tree and its surrounding vegetation in the PHOLIAGE-model**. Characteristics of both tree and vegetation can be varied.

We assume a tree with specific crown dimensions placed in a vegetation stand with a specific canopy height (Figure 1). The length to width ratio of the ellipsoid crown can be varied such that vertically elongated, spherical, and flatter broader crowns can be considered. The canopy of the surrounding vegetation can be fully closed and thus encompass and even overtop the tree, or it can be virtually opened up as a circular gap around the tree. Characteristics of the tree and surrounding vegetation can be varied. With a technique called ray-tracing (Pearcy and Yang, 1996; Rohrig et al., 1999; Bartelink, 2000) the amount of light absorbed in each point in the tree's crown is calculated and subsequently the photosynthetic rate for each point is calculated. An integration over all points is made to calculate whole crown light capture and photosynthetic rate.

Dividing instantaneous whole crown light capture (μmol s^−1^, Equation S12a) and whole crown photosynthetic rates (μmol s^−1^, Equation S1) by leaf area, gives the mean light capture per unit leaf area (Φ_area_ in μmol m^−2^s^−1^) and photosynthetic rates per unit leaf area (*P*_area_ in μmol m^−2^s^−1^). This was done to correct for differences in plant size, i.e., a large plant may exhibit a greater total photosynthesis but this may not necessarily be the result of its leaves being positioned more favorably relative to the light gradient or because of a more efficient leaf physiology, which would be reflected in the values of Φ_area_ and *P*_area_ (Anten and Hirose, 2003; Selaya and Anten, 2010).

**Figure 2:**
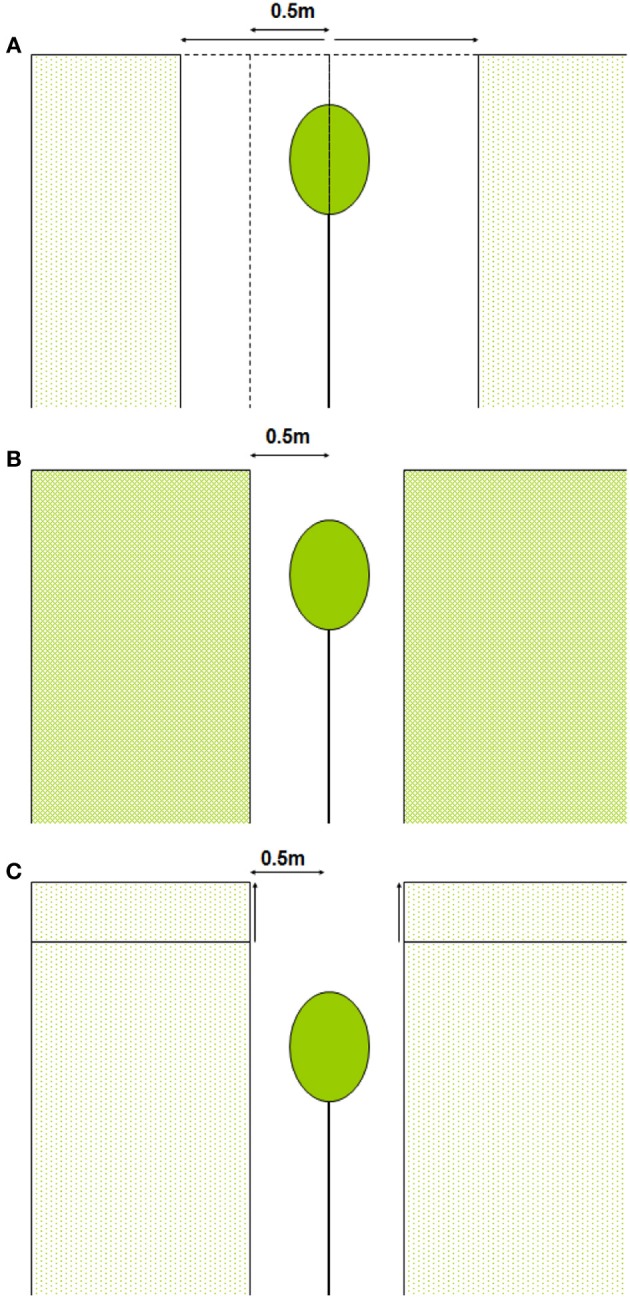
**(A–C)** Schematic presentation of model simulations. In **(A)** vegetation is removed around the individual within a variable radius, in **(B)** the LAI of the surrounding vegetation is increased and in **(C)** the vegetation height is increased (leaf area density remains constant).

We calculated whole-canopy light capture and photosynthetic rates of each individual control tree and of liberated trees immediately after removal of the vegetation in the 1.5 y/o stand. Next, we performed four model simulations. First, we simulated the effect of an increasing gap radius in which vegetation was virtually removed (Figure 2A). Second, we simulated the effect of a vegetation removal event (gap radius = 0.5 m) in a vegetation stand with an increasing LAI (Figure 2B). Third, the effect of surrounding vegetation height was simulated in a vegetation removal event (gap radius = 0.5 m; leaf area density of the vegetation was kept constant while height varied) (Figure 2C). Fourth, we simulated vegetation removal events with variable gap radii in three successional forest stands (i.e., stands with different LAI and height, see Table 1).

**Table 1:** **Characteristics of successional vegetation stands in a Vietnamese forest (average values ± *SE*)**.

	**Stand I**	**Stand II**	**Stand III**
Age (year)	~0.5	~1	~1.5
Mean LAI (m^2^m^−2^)	3.03 ± 1.74	5.46 ± 0.91	3.73 ± 0.75
Mean height (m)	0.61 ± 0.18	0.99 ± 0.31	1.40 ± 0.41

*Data are taken from Van Kuijk et al. (2008)*.

In the first, second and third simulation we used average values for tree height, crown dimensions, leaf area, leaf angle distribution and leaf nitrogen content per species based on all existing individuals of a species in the 1.5 y/o stand. We also assumed average characteristics (LAI, canopy height and leaf angle distribution) of the surrounding vegetation as measured in the study. This was done to analyze the effect of an increasing gap radius, vegetation LAI and vegetation height on species with different dimensions, leaf area and leaf nitrogen content. In the fourth simulation we performed virtual vegetation removal experiments with variable gap radii for individual trees within their surrounding vegetation based on actual field data as measured in a previous study (Van Kuijk et al., 2008). To estimate the extent to which the effect of liberation would depend on the successional age of the vegetation, we simulated tree liberation for three successional stands (0.5, 1, and 1.5 years after field abandonment) whereby the characteristics of these stands and target plants therein were taken from Van Kuijk et al. (2008).

### Statistical analysis

Plots were created after the slash and burn treatment with the sole purpose to be able to trace back the saplings in following measuring periods. However, in this set-up plot-effects might occur. Therefore, analyses were done with Linear Mixed Effects Model (in results section abbreviated as MM).

The effect of species on the average values of light capture, photosynthetic rates and growth were determined by identifying these variables as dependent and species as independent factor. When designing the statistical model, species was the sole fixed effect, including intercept. For random effect an intercept was included for the subject plot number, but no model for random effects was designed. No *post-hoc* tests were available but most between-species differences could be deduced from the parameter estimates.

The relation between observed growth (biomass in g day^−1^) and calculated photosynthetic rate was analyzed with linear regression and the difference in slopes was analyzed with ANCOVA.

## Results

### Vegetation removal in the 1.5 y/o stand

The average height of the vegetation in the 1.5 y/o stand was 1.4 m and the LAI was on average 3.73 (Table 1). Species differed in height but were on average lower than the surrounding vegetation (Table 2). Average growth characteristics differed between liberated and control trees after liberation (Table 3) with the liberated trees growing less in height, but more in biomass.

**Table 2:** **Average characteristics of tree species in a 1.5 y/o stand (average values ± *SE*)**.

**Plant characteristics**	**Height**	**Biomass**	**Leaf area**	**Leaf angle fractions**			**Crown vol**.	**Crown l:w**	**Lad**	**N_o_**	**P_max_–N_area_ relation**	**R_d_**	**Quantum yield**
**Species**	**(m)**	**(g)**	**(m^2^)**	**15**	**45**	**75**	**(m^3^)**		**(m^2^ m^−3^)**	**(mmol m^−2^)**	**Slope**	**(mmol m^−2^s^−1^)**	**(mmol C PPFD^−1^)**
*Mallotus microcarpus*	1.26 ± 0.07	52.70 ± 8.48	0.29 ± 0.02	0.23	0.48	0.29	0.04 ± 0.00	1.18 ± 0.03	6.72 ± 0.27	94.28 ± 1.25	0.17 ± 0.05	1.04 ± 0.02	0.03 ± 0.00
*Mallotus paniculatus*	0.94 ± 0.01	12.22 ± 0.56	0.06 ± 0.00	0.13	0.41	0.46	0.01 ± 0.00	2.00 ± 0.03	4.94 ± 0.15	79.33 ± 0.84	0.10 ± 0.05	0.24 ± 0.01	0.03 ± 0.00
*Macaranga denticulata*	1.07 ± 0.03	27.22 ± 1.17	0.11 ± 0.00	0.37	0.40	0.24	0.03 ± 0.00	1.42 ± 0.02	4.35 ± 0.15	82.52 ± 0.72	0.18 ± 0.06	0.53 ± 0.01	0.04 ± 0.00

*For the data on biomass allocation n = 20, 31, and 31 individuals for M. microcarpus, M. paniculatus and M. denticulata respectively*.

*For the photosynthesis data n = 15–23, 15–28, and 16–17 leaves for M. microcarpus, M. paniculatus and M. denticulata respectively*.

*Values are calculated based on individuals in the 1.5 y/o stand (data were also taken from Van Kuijk et al. (2008). Leaf angle fractions are divided into three classes indicated by 15, 45, and 75 degrees, crown l:w is the ratio between the length and the width of the crown, lad is leaf area density, No is maximum nitrogen content of the leaves, Rd is dark respiration*.

**Table 3:** **Average growth characteristics of liberated and control trees 174 days after liberation (average values ± *SE*)**.

**Species**	**Average height liberated trees**	**Average height control trees**	**Height growth liberated trees**	**Height growth control trees**	**Average biomass liberated trees**	**Average biomass control trees**	**Biomass growth liberated trees**	**Biomass growth control trees**
	**(cm)**	**(cm)**	**(cm/day)**	**(cm/day)**	**(g)**	**(g)**	**(g/day)**	**(g/day)**
*Mallotus microcarpus*	180.00 ± 9.51	184.82 ± 9.27	0.31 ± 0.01	0.41 ± 0.02	121.41 ± 12.00	93.56 ± 13.79	0.35 ± 0.03	0.28 ± 0.04
*Mallotus paniculatus*	121.00 ± 1.58	139.13 ± 2.07	0.19 ± 0.01	0.28 ± 0.01	56.26 ± 4.46	41.12 ± 2.26	0.25 ± 0.02	0.17 ± 0.01
*Macaranga denticulata*	133.14 ± 3.13	153.65 ± 2.72	0.16 ± 0.01	0.26 ± 0.00	68.57 ± 3.79	54.97 ± 2.87	0.25 ± 0.02	0.16 ± 0.01

*For the liberated trees n = 9, 15, and 15 for M. microcarpus, M. paniculatus and M. denticulata respectively*.

*For the control trees n = 11, 16, and 16 for M. microcarpus, M. paniculatus and M. denticulata respectively*.

**Figure 3:**
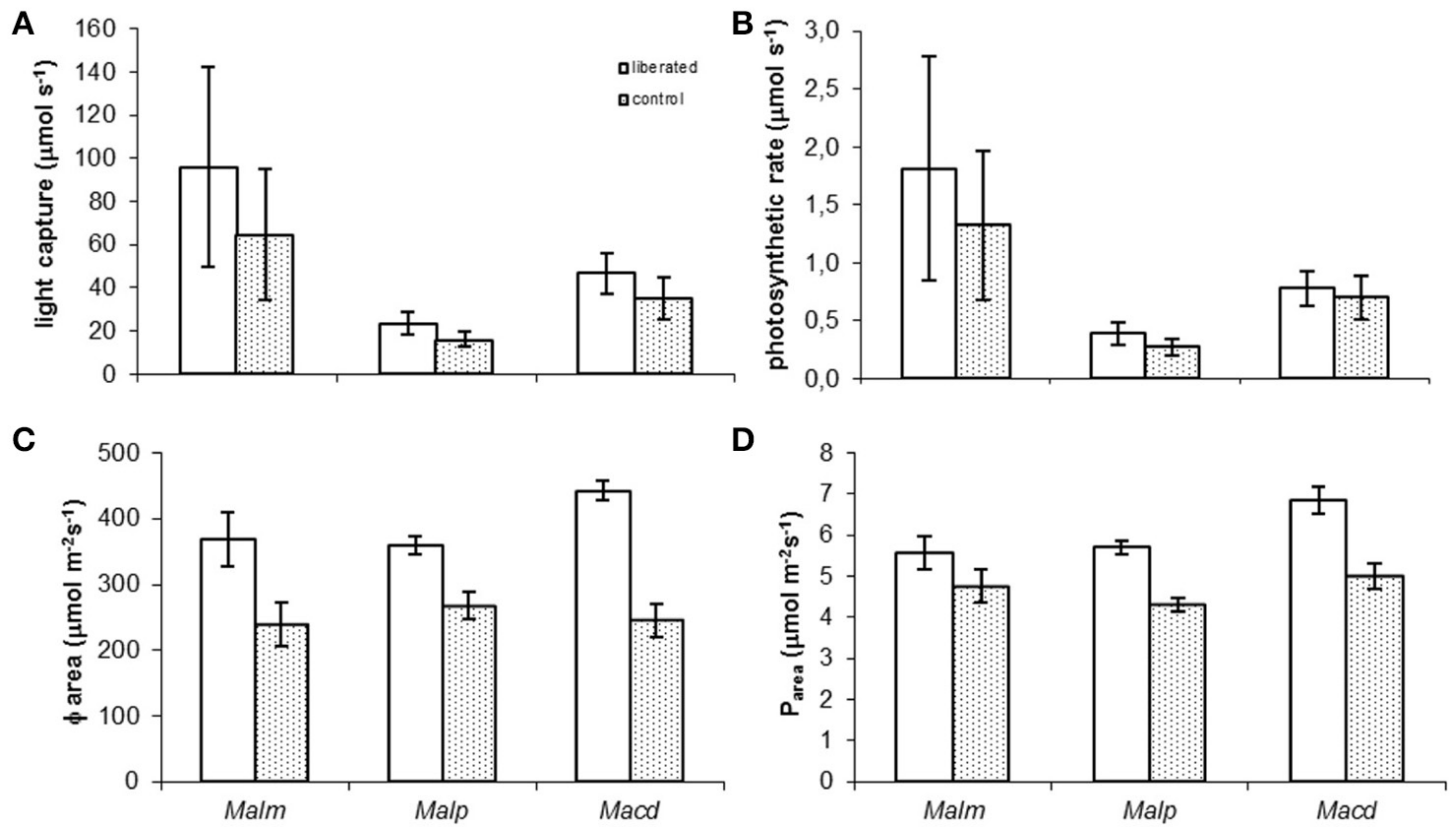
**(A–D)** Mean values of whole canopy light capture and photosynthetic rate of control and liberated plants immediately after vegetation removal (gap radius = 0.5 m) in a 1.5 y/o secondary forest stand in Vietnam (the horizontal light intensity on top of the canopy was set to 1000 μmol m^−2^s^−1^). Note that values in **(A,B)** are smaller than the rate per m^2^ **(C,D)** as the leaf area of the trees is on average less than 1 m^2^. Abbreviations:*Malm, Mallotus microcarpus; Malp, Mallotus paniculatus; Macd, Macaranga denticulata*. Bars denote standard error.

Model calculations showed that in all species liberated individuals on average tended to have higher absolute whole-canopy light capture rates (μmol s^−1^) (the horizontal light intensity on top of the canopy was set to 1000 μmol m^−2^s^−1^) and photosynthetic rates (μmol s^−1^) immediately after removal of vegetation than the control individuals (Figures 3A,B) but none of these differences were significant (MM, *p* > 0.05). Liberated plants had significant higher light capture per unit of leaf area (Φ_area_) and photosynthetic rate per unit of leaf area (*P*_area_) (Figures 3C,D) (MM, *p* < 0.05) than control plants, except for *P*_area_ of *Mallotus microcarpus* (MM, *p* = 0.133).

**Figure 4:**
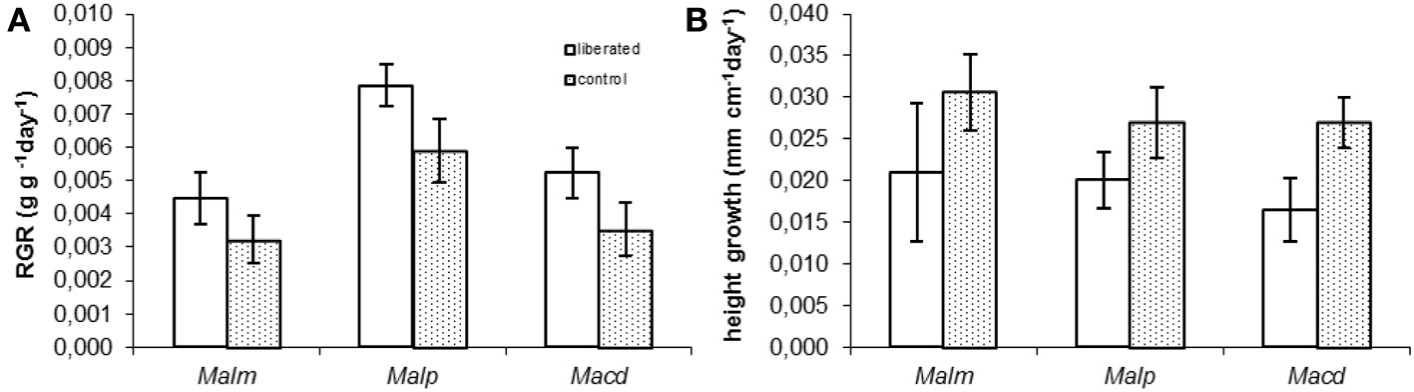
**(A,B)** Measured above ground Relative Growth Rate (RGR) and relative height growth rate of control and liberated plants in 174 days after vegetation removal (gap radius = 0.5 m). For abbreviations see Figure 3. Bars denote standard error.

Relative growth rates in terms of biomass (RGR) and height were measured in the period following the removal of the surrounding vegetation (Figure 4). Vegetation removal tended to result in a higher RGR (Figure 4A), but this was only significant for *Mallotus paniculatus* (MM, *p* = 0.043). Height growth tended to be lower for liberated plants (Figure 4B), but this was only significant for *Macaranga denticulata* (MM, *p* = 0.036).

**Figure 5:**
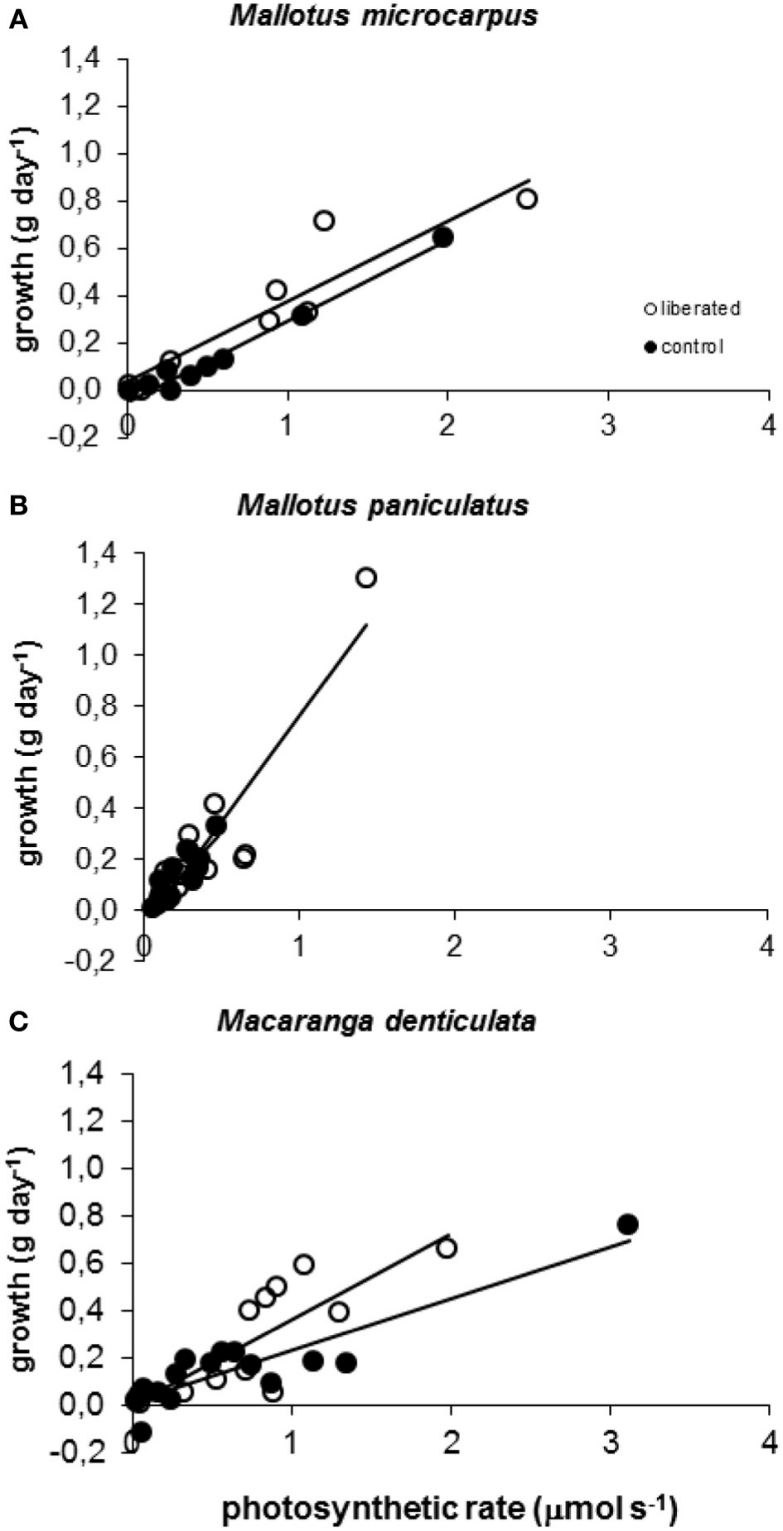
**(A–C)** Correlation between observed above ground biomass growth in 174 days after vegetation removal and calculated values of whole canopy photosynthetic rates immediately after vegetation removal obtained with the PHOLIAGE model. All correlations are significant (MM, *p* < 0.01) and *r*^2^ values varied from 0.6928 to 0.9744.

Model calculations of instantaneous absolute photosynthetic rates immediately after removal of vegetation, were significantly correlated to above ground mass growth (g day^−1^) for all species in both the control and liberation treatment in the period following removal (Figure 5). The slopes of these relationships did not differ significantly between the control and liberation treatment (MM, *p* > 0.05), indicating that the model was well able to predict the effect of liberation on growth. Slopes were higher for *Mallotus paniculatus* than for the other species (MM, *p* = 0.011).

### Model simulation: variation in gap radius

**Figure 6:**
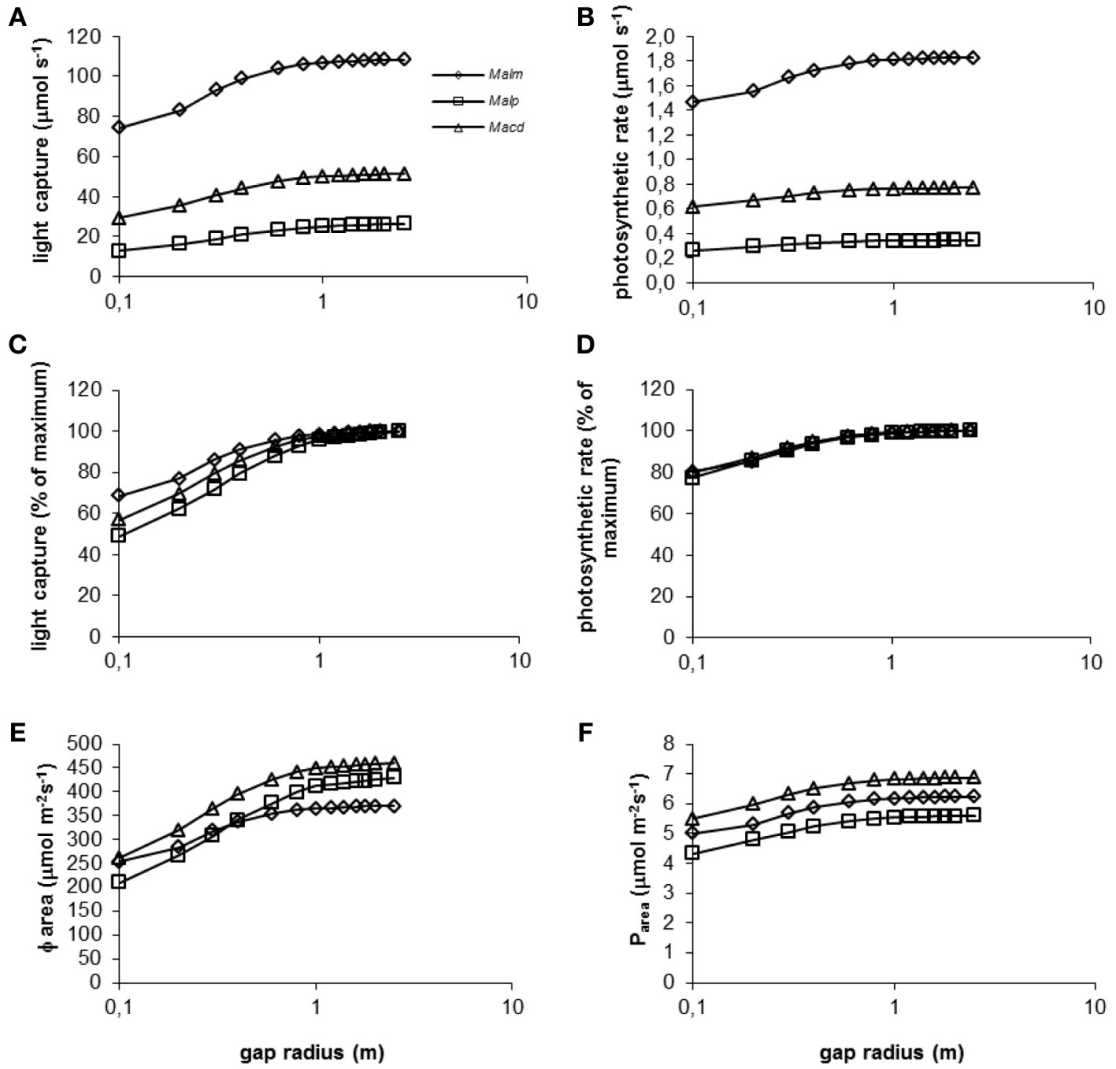
**(A–F)** Model simulations of the effect of gap radius (log scale) on whole canopy light capture and photosynthetic rate of species in a 1.5 y/o secondary forest stand. Mean parameter values were used for each species and treatment. For abbreviations see Figure 3.

Values of absolute light capture and photosynthetic rates increased with increasing gap radius and reached near-maximum values at a gap radius of approximately 1 m (Figures 6A,B). *Mallotus microcarpus* captured the largest amount of light and had the highest photosynthetic rate while *Mallotus paniculatus* captured the least light and had the lowest photosynthetic rate. This corresponded with species height and leaf area (Table 2). *Mallotus microcarpus* was the tallest species, the top of its crown reached on average 90% of the surrounding vegetation height, and it had the most leaf area. *Mallotus paniculatus* was the shortest species with a mean height of 67% of the surrounding vegetation height, and it had the smallest leaf area. *Macaranga denticulata* showed intermediate values for light capture, photosynthetic rates, height, and leaf area.

*Mallotus microcarpus* reached maximum values of light capture (as a percentage of light capture at a gap radius of 2.5 m, which showed maximum values of light capture for all species) before the other species did (Figure 6C). This was related to the crown shape of the species and species height. *Mallotus microcarpus* had the smallest crown length compared to its crown width (Table 2) and it was positioned relatively high in the canopy. Thus, with increasing gap radius, the bottom part of its crown will be capturing maximum light levels sooner than a crown that is placed lower in the vegetation and has a longer crown length compared to its width like that of *Mallotus paniculatus*. *Macaranga denticulata* had an intermediate crown size and was positioned at intermediate height in the vegetation and the simulation line in Figure 6C fell between that of the other species.

Photosynthetic rate as percentage of the maximum photosynthetic rate was approximately equal for all species at all gap radii (Figure 6D). The discrepancy between Figure 6C and Figure 6D can be explained by the photosynthetic characteristics of the species (Table 2). *Mallotus microcarpus* could achieve high levels of maximum photosynthetic rates (high *N_o_* and high slope of the *P*_max_ - *N*_area_ relation) and had a high dark respiration (*R_d_*). *Mallotus paniculatus* on the other hand, was not able to achieve such high levels of maximum photosynthetic rates and its dark respiration was low, which is favorable in low light conditions. *Macaranga denticulata* could also achieve relatively high maximum photosynthetic rates and had intermediate *R_d_*. Thus, the tallest species, *Mallotus microcarpus*, was best able to keep up with the surrounding vegetation height, but was also most light-demanding.

With increasing gap radius, Φ_area_ increased for all species (Figure 6E). The smaller increase in *Mallotus microcarpus* can be explained by its relatively short crown and its position relatively high in the canopy. Many leaves already experience high light levels and an increase in gap radius will increase Φ_area_ but not as much as for a tree with a more elongated crown positioned lower in the canopy. The relatively low value of maximum Φ_area_ for *Mallotus microcarpus* is the consequence of its greater leaf area density compared to the other species (Table 2). This increases self-shading.

*Mallotus paniculatus* had the lowest mean photosynthetic rate per unit leaf area (Figure 6F). This was related to its relatively low maximum photosynthetic rate. *Macaranga denticulata* showed the highest *P*_area_because of its high maximum photosynthetic rates and high Φ_area_.

### Model simulation: variation in LAI and vegetation height

**Figure 7:**
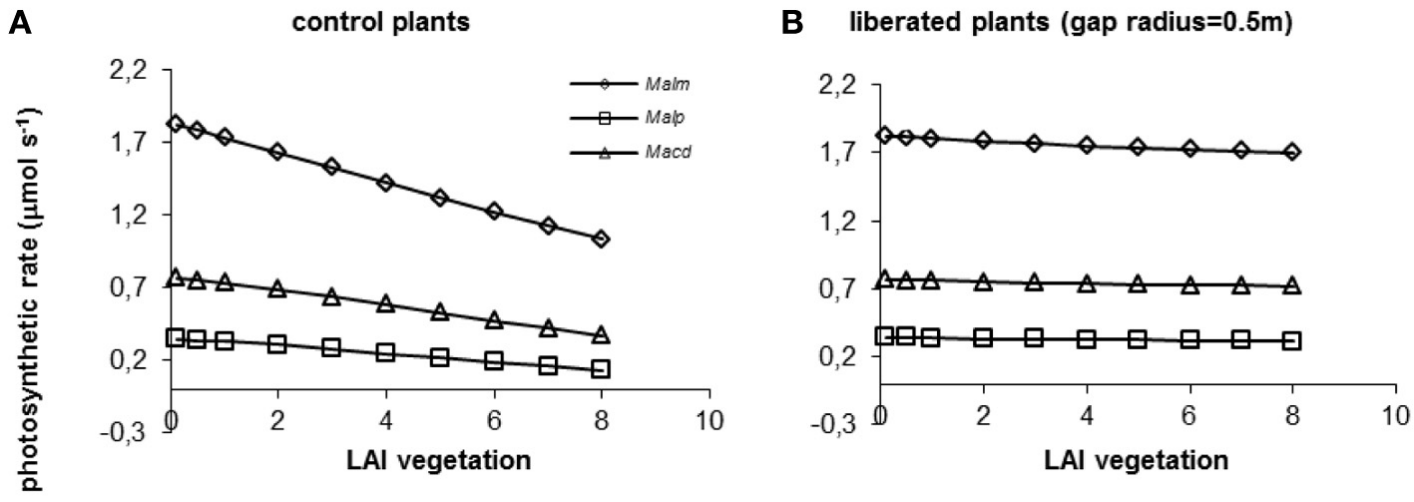
**(A,B)** Model simulations of the effect of LAI of the surrounding vegetation on whole canopy photosynthetic rates of species in a control situation (surrounding vegetation intact) **(A)** and liberated plants **(B)** in a 1.5 y/o secondary forest stand. Mean parameter values were used for each species and treatment. For abbreviations see Figure 3.

When the surrounding vegetation was left intact, photosynthetic rates declined with increasing LAI for all species (Figure 7A). The decline was steepest for the species highest in the canopy, *Mallotus microcarpus*, because in absolute terms light availability diminishes more strongly higher in the canopy than lower down, and this species had higher respiration. When vegetation was removed around the individual trees in a radius of 0.5 m, the effect of its LAI on photosynthetic rates was almost completely diminished (Figure 7B).

**Figure 8:**
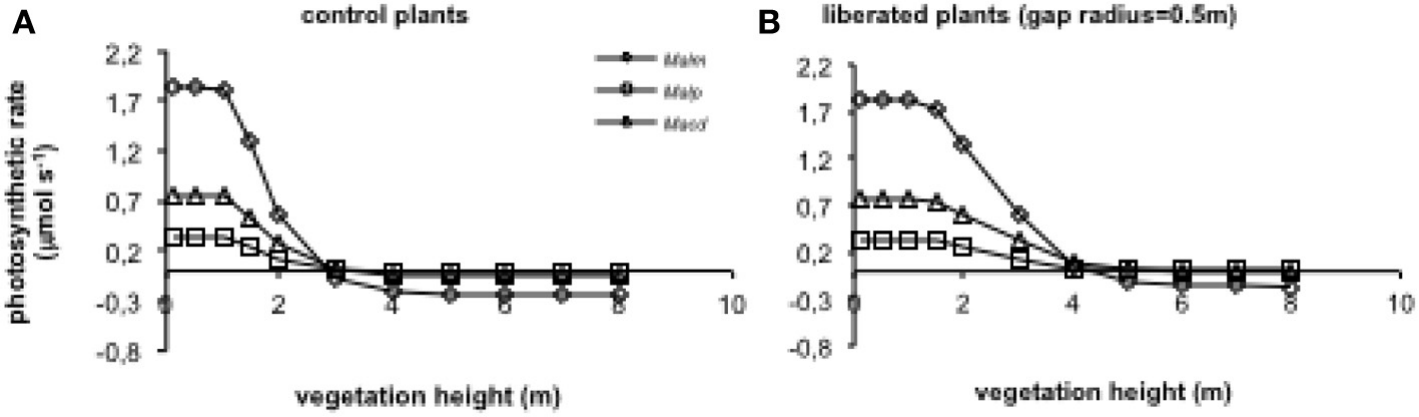
**(A,B)** Model simulations of the effect of height of the surrounding vegetation on whole canopy photosynthetic rates of species in a control situation (surrounding vegetation intact) **(A)** and liberated plants **(B)** in a 1.5 y/o secondary forest stand (LAI was changed with vegetation height so that leaf area density remained constant). Mean parameter values were used for each species and treatment. For abbreviations see Figure 3.

When vegetation height was increased (for average vegetation characteristics see Table 1), photosynthetic rates declined in the control and the liberation treatment (Figure 8), but the effect for the control plants was stronger than for the liberated plants. *Mallotus microcarpus* reached negative photosynthesis values before the other species did. The sudden decline in photosynthetic rate indicated the moment the height of the surrounding vegetation exceeded that of the target trees.

### Model simulation: vegetation removal in successional stands

**Figure 9:**
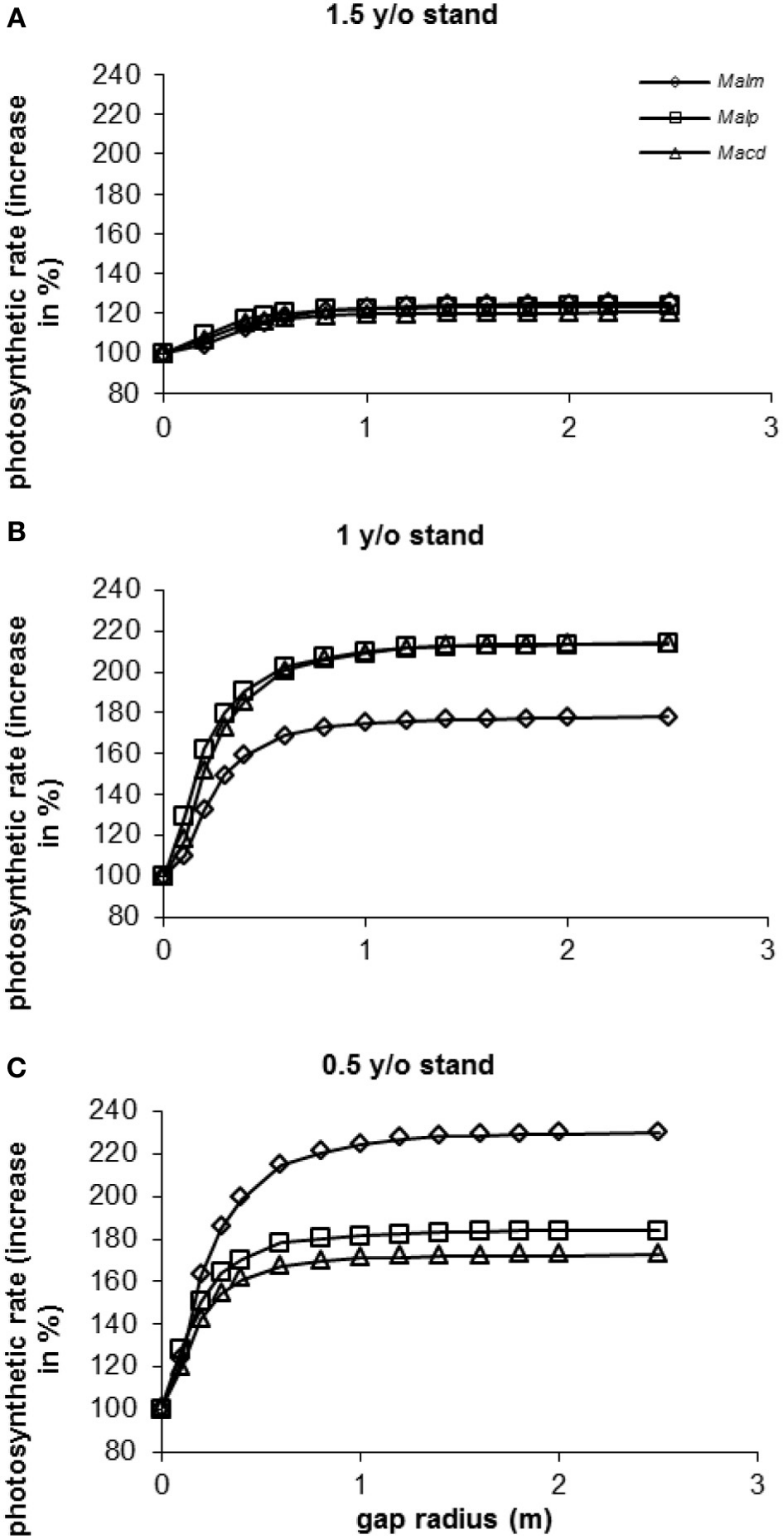
**(A–C)** Model simulations of the effect of gap radius on whole canopy photosynthetic rates of species in three successional vegetation stands of the same forest. Simulations were done for individual trees [data were taken from Van Kuijk et al., 2008]. For abbreviations see Figure 3.

In all stands and for all species photosynthetic rate (as a percentage of photosynthetic rate in the control situation: gap radius = 0 m) increased with increasing gap radius (Figure 9). In the 1.5 y/o stand the effect of vegetation removal was not as great as in the 1 and 0.5 y/o stands. In the 1.5 y/o stand trees were on average closer to the top of the vegetation than in the younger stands (see Van Kuijk et al., 2008). Average photosynthetic rates in the 1.5 y/o stand were therefore closer to the maximum values than in the same individuals in the younger stands.

In the 1 y/o stand (Figure 9B) it seems as if vegetation removal affected photosynthetic rates of *Mallotus microcarpus* less than those of *Mallotus paniculatus* and *Macaranga denticulata*. In the 0.5 y/o stand (Figure 9C) the opposite seemed to occur. In the 0.5 y/o stand all species had approximately the same height respective to the vegetation height (results not shown). *M. microcarpus* had a two-fold higher leaf area and a lower leaf area density than the other species (results not shown), resulting in a higher potential photosynthetic rate. In the 1 y/o stand *M. microcarpus* had the highest height respective to the vegetation height (results not shown), so it was closer to maximum levels of photosynthesis than the other species. Therefore, an increase in gap radius had less effect.

When calculating the increases in photosynthetic rates as percentage of the maximum photosynthetic rates (at a gap radius of 2.5 m), differences between species and stands were small (90–96% with a gap radius of 0.5 m and 98–99% with a gap radius of 1 m, depending on species and stand).

## Discussion

### Species responses to liberation

Our model calculations indicated that removal of surrounding vegetation around target trees in a young secondary forest stand was beneficial in terms of light capture and photosynthetic rate. The field measurements showed that species in this study responded slightly different to the treatment depending on their morphological and physiological characteristics. Model calculations showed that the responses were also dependent on the characteristics of the surrounding vegetation. There were strong correlations between calculated photosynthetic rates and observed growth, indicating that the model presented in this paper was well able to predict the growth of trees in early successional forests. These relationships did not differ significantly for the liberated and control plants. This indicates that the model was capable of predicting the magnitude of liberation effects on tree growth. This model can therefore be utilized to understand how tree species traits and the pattern and timing of vegetation removal together drive the effects of tree liberation. As such it is a first important step toward developing a predictive tool in designing experiments and management scenarios to improve the effectiveness of this intervention measure.

In the 1.5 y/o stand vegetation removal in a gap radius of 0.5 m resulted in higher light capture and higher photosynthetic rate per unit leaf area and a tendency toward higher biomass growth and reduced height growth of the trees as compared to the trees in the control situation. Similar results were obtained by Collet et al. (1998) in an oak plantation. They attributed these differences in growth to the reduction of belowground competition between neighboring plants and the target trees. However, the close correspondence between predicted effects of liberation by the model, which includes only aboveground competition, and the measured effects on growth, suggests that light competition played a more prominent role in our study.

The reduced relative height growth in liberated plants was considerable, 25–40%, and seems counterintuitive since biomass increment was stimulated. Plants typically respond to the proximity of neighbor plants through increased stem elongation, reduction in stem diameter and thus a greater plant height per unit mass (Smith, 1982; Selaya et al., 2007). This response, commonly denoted as the shade avoidance syndrome (Smith, 1982) tends to be stronger in early than in late successional tree species (Gilbert et al., 2001). Liberation, removal of neighbor plants, may thus inhibit stem elongation. This could potentially have a negative impact on the competitive ability of liberated trees if the surrounding vegetation regrows after liberation and gradually fills the gap.

Near-maximum values of light capture and photosynthetic rates were reached at a gap radius of approximately 1 m. Additional cutting in a bigger radius would hardly increase light capture and photosynthetic rates. Korpelainen et al. (1995) found that planted trees showed no additional growth in strips wider than 2 m in a 3 m high tropical forest. The radius (or width in case of a strip) at which near maximum light levels for growth are reached depends on the vegetation height relative to that of the target tree and will thus be different for every forest stand (Korpelainen et al., 1995; Pena-Claros et al., 2002). The optimal radius for a stand could be determined with the model presented here.

The degree to which light capture of plants increased with increasing gap radius differed considerably between species. This could be related to interspecific differences in morphological traits such as leaf area, tree height (i.e., the crown's position in the canopy) and crown dimensions. Crown architecture determines the display of leaves, light interception, and thus carbon acquisition (Bongers and Sterck, 1998). In our study leaf area density appeared to influence light capture per unit leaf area. A high leaf area density results in increased self-shading within the crown and this reduces light capture. Shaded leaves do not necessarily have negative carbon balances but self-shading does lower whole plant carbon gain (Pearcy and Yang, 1996; Sterck et al., 2003). Differences in physiological traits between species explained the response of the photosynthetic rates to increases in gap radius, and to increases in the LAI and height of the surrounding vegetation. *Mallotus paniculatus* plants had lower photosynthetic capacities but also lower rates of dark respiration than the other species, and this favored their net carbon gain at low, but not at high light. Thus, while this species exhibited a greater increase in light capture per unit leaf area with increasing gap radius, it did not similarly show a greater increase in net photosynthesis. However, a low dark respiration (incorporated in the model) is often correlated to low respiration of other plant parts (not in the model), which might explain the greater carbon use efficiency, i.e., the larger ratio between growth and photosynthesis of *Mallotus paniculatus* as compared to the other two species. We therefore recommend that future model analyses should take respiration of non-photosynthetic tissue into account.

Above mentioned results show that even closely related species, can differ considerably in their optimal light conditions for growth, consistent with previous findings (Ramos and del Amo, 1992; Korpelainen et al., 1995; Montagnini et al., 1997; Dupuy and Chazdon, 2006). In our study it appeared that the species that was best able to keep up with the growing surrounding vegetation was the most light-demanding one. Slower growing species grow in increasingly darker environments since they lack the ability to grow tall rapidly, however, they are also less light-demanding and respond less strongly to increasing light levels. As a result of these two opposing mechanisms the effects of increasing gap radius can be rather similar for species with different light requirements (see Figure 9A) growing at different light levels.

### Enhancing forest recovery through liberation

When trees are no longer hampered in growth by the surrounding vegetation, their biomass allocation pattern seems to change. Less biomass is invested in height because the need to grow tall is reduced and instead trees create denser canopies (Collet et al., 1998). This is important in younger successional stands since shrubs, even though they are architecturally constrained to grow tall, can still keep up with tree height and thus overtop trees in such young stands. A denser tree crown will increase the shading out of the shrubs and grasses growing underneath the tree's crown. Once the tree has overtopped and (partly) shaded out the surrounding vegetation, it will be hampered considerably less in growth.

Vegetation removal experiments in forests generally result in increased growth of the liberated trees (Collet et al., 1998; Finegan et al., 1999) but they have not always been successful because in some cases vegetation surrounding trees appeared to facilitate tree recruitment by changing soils conditions (Vieira et al., 1994; Aide et al., 1996; Li et al., 1999). The degree of success of a vegetation removal event depends on many factors such as site characteristics (soil moisture content and nutrient availability) (Putz and Canham, 1992; Li et al., 1999), the light requirement of the target species (Dupuy and Chazdon, 2006; Petritan et al., 2007), the type of plants in the surrounding vegetation (Vieira et al., 1994; Holl, 1998), the number of removal events (De Graaf et al., 1999; Pariona et al., 2003) and the timing of the intervention (Fuhr et al., 2001). Our model yields insights into how the effects of vegetation removal can be mediated by the characteristics of the target species and the vegetation, the radius in which vegetation is removed and the timing of removal events. For this specific forest we showed that the greatest effects of vegetation removal are realized if the vegetation is removed within the first year of stand development. We also demonstrated that increasing the gap radius from 0.5 to 1 m resulted in a relatively small increase in whole-plant photosynthesis. Such an increase in gap radius, however, will require more labor and thus incur more costs. These resources could be spared or could be used to do a subsequent 0.5 m liberation later in time (though we did not test the effects repeated liberation). Even though factors such as soil and climate characteristics are not incorporated in the model, the simulation of vegetation removal in different successional stands is an appropriate practical example that shows the potential use of the model for decisions in the field.

### Model application

The PHOLIAGE model is not the only model that calculates tree photosynthetic rates but is unique in that it is used to approach a practical problem in restoration ecology. In very early stages of tropical forest succession, such as in this study, the PHOLIAGE model approach proved to be effective in predicting growth and we believe it is therefore a first important step toward a predictive tool for estimating effects of tree liberation and similar forest management practices. However, the model still contains a number of simplifications. First interaction for belowground resources is not considered. Liberation does not only entail release from light competition but also partial release from belowground competition. In our case the vegetation surrounding trees was mostly composed of perennial grasses, whereby removal of aboveground parts probably reduces root functioning. Thus, liberated trees not only experienced more light but probably also greater availability of soil resources, though demand for soil resources probably increased. Second, the model did not consider growth dynamics of the study tree (including respiration of non-leaf tissue and shifts in biomass allocation). For example, liberated trees may increase biomass allocation to roots and this could modify their response. These effects should be accounted for in future models to improve generality of application to more situations in tropical regions.

Model parameters are easy to gather relative to some other more detailed growth models (Pearcy and Yang, 1996; Sterck and Schieving, 2007), but we realize it still involves some physiological measurements such as photosynthesis which may not always be available. In those cases and depending on the tree species in question reliable data might be available in databases of species functional traits (Wright et al., 2004). However, care needs to be taken as these traits are plastic and differ genetically within species.

In this study light capture and photosynthesis were calculated instantaneously. If the model were to be applied in non-tropical areas, the larger variation in light climate (during the day and during the year, and related to geographical position) would need to be taken into account. This affects the amount of light a plant receives and consequently plant growth. Also rainfall and soil characteristics may affect growth. If the model is to be used in ecosystems that are located far from the equator (for instance in temperate zones), or in systems that experience strong seasonal effects, or where water or soil factors greatly affect plant growth, it will need to be extended.

### Conflict of interest statement

The authors declare that the research was conducted in the absence of any commercial or financial relationships that could be construed as a potential conflict of interest.
