# Supplementary material for: Stimulating seedling growth in early stages of secondary forest succession: a modeling approach to guide tree liberation
Source: Front Plant Sci. 2014 Jul 18;5:345. doi: 10.3389/fpls.2014.00345 (PMC4102908; doi:10.3389/fpls.2014.00345)
Supplement: Supplementary file 1 [file Presentation1.PDF]

Supplementary material

Stimulating seedling growth in early stages of secondary forest succession: a modeling approach to guide tree liberation.

Marijke van Kuijk[1*], Niels P.R. Anten[1,2], Roelof J. Oomen[1] and Feike Schieving[1]

[1]Institute of Environmental Biology, Department of Biology, Utrecht University, Utrecht, the Netherlands
[2]Centre for Crop Systems Analysis, Wageningen University, Wageningen, the Netherlands

[*]Dr. Marijke van Kuijk
Institute of Environmental Biology
Department of Biology
Utrecht University
PO Box 80084
3508 TB Utrecht
The Netherlands
m.vankuijk@uu.nl

PHOLIAGE-model description

In this section the PHOLIAGE-model is described in detail. First it is explained how integration over an ellipsoid crown is done in order to calculate whole crown light absorption and photosynthetic rate. Next we explain how the photosynthetic rate per volume element of the crown is calculated and how this is determined by light absorption in the volume element and leaf nitrogen content. Finally we show how the light intensity for each point in the crown can be found by ray-tracing. Here path lengths of the light beam through the crown and the surrounding vegetation are taken into account. Note that we start with how the total photosynthetic rate of the plant is to be interpreted as a sum (an integral) of rates over the crown, and end with a formalisation of the light coming from the sky.

1 The model tree and its surrounding vegetation

We assume a target tree with an ellipsoid shaped crown placed in a vegetation stand (see Fig. 1 in the manuscript). All positions in and around the ellipsoidal crown are given in terms of Cartesian coordinatization, with the origin in the centre of the ellipsoid. The shape of the ellipsoid is characterised by the lengths of the semi-axes *a*, *b* and *c* in the *x*-, *y*- and *z*-direction, with the *z*-axis pointing towards the zenith.

The tree crown is surrounded by vegetation with infinite horizontal extensions. The vertical dimensions of the vegetation are given by top height h_t and bottom height h_b. The tree and the surrounding vegetation are completely separated in terms of leaf distribution. The vegetation around a target tree can be opened up as a cylinder with the *z*-axis of the cylinder coinciding with the *z*-axis of the ellipsoid. The radius of the cylinder (r_{gap}) can have any non-negative value and is not limited by the *a*- or *b*-axes of the ellipsoid. The top and bottom positions of the ellipsoid (with $z=-c$ and $z=c$) can have any position with respect to the surrounding vegetation. For both the crown and the surrounding vegetation, leaf area density and leaf angle distribution are assumed to be homogeneous.

2 Whole crown photosynthetic rate and photon absorption rate

The net whole crown photosynthetic rate is calculated by integrating the net photosynthetic rate per unit volume over the crown. P_n denotes the net photosynthetic rate per unit volume at a point **p** within the ellipsoidal crown. The total net photosynthetic rate of the crown P_E (in μmol s^{-1}) is given by the integral:

$$P_E = \int_{ellipsoid} P_n(\mathbf{p})dV \qquad (1)$$

To simplify the integration the ellipsoid is related to a unit sphere, meaning that every point **p**' in the unit sphere is related to a point **p** in the ellipsoid by:

$$(p_x, p_y, p_z) = (ap'_x, bp'_y, cp'_z) \qquad (2)$$

The photosynthetic rate per unit volume $P_n'(\mathbf{p}')$ in the unit sphere is related to the photosynthetic rate $P_n(\mathbf{p})$ in the associated ellipsoid by:

$$\mathbf{p}' \rightarrow P_n'(\mathbf{p}') = P_n(ap'_x, bp'_y, cp'_z) = P_n(\mathbf{p}) \quad (3)$$

For the net crown photosynthetic rate we now find:

$$P_E = abc \int_{sphere} P_n'(\mathbf{p}')dV' \quad (4)$$

When expressing the Cartesian coordinates in terms of cylindrical coordinates this results in:

$$P_E = abc \int_{-z}^{+z} \int_{0}^{R(z')} r' \int_{0}^{2\pi} P_n'(\mathbf{p}')d\psi' dr' dz' \quad (5)$$

with

$$\mathbf{p}' = (r'\cos\psi', r'\sin\psi', z')$$

$$R(z') = \sqrt{1 - z'^2}$$

and

$$z' \in (-1, 1)$$

Net crown photon absorption rate is calculated similarly, by substituting P_E with I_E and P_n with I_n (for P_n and I_n see next sections).

3 Photosynthetic rate per unit volume

Net photosynthetic rate per unit crown volume P_n (μmol $m^{-3}s^{-1}$) is calculated by integrating the product of net photosynthetic rate per unit leaf area for leaves with normal $\mathbf{d}_L$ and the fraction of the total leaf area in direction $\mathbf{d}_L$ per steradian f^{Ω}, over all positive (faced upwards) leaf angles Ω_L (steradian):

$$P_n(\mathbf{p}) = \int_{\Omega_L} f^{\Omega}(\mathbf{d}_L) P_L(\mathbf{p}, \mathbf{d}_L) d\Omega_L \quad (6a)$$

Using spherical coordinates, $P_n(\mathbf{p})$ can be written as:

$$P_n(\mathbf{p}) = \int_{0}^{\frac{\pi}{2}} \sin\vartheta_L f^{\Omega}(\vartheta_L) \int_{0}^{2\pi} P_L(\mathbf{p}, \vartheta_L, \psi_L) d\psi_L d\vartheta_L \quad (6b)$$

in which f^{Ω} is assumed to be independent of the azimuth angle and leaf normals are assumed to be directed into the upper hemisphere.

Net photosynthetic rate per unit leaf area P_L (μmol $m^{-2}s^{-1}$) in point **p** for light direction **d** is calculated from net photosynthetic rate per unit leaf area p_L which is a function of net photon absorption rate per unit leaf area I_L and leaf nitrogen content N:

$$P_L(\mathbf{p}, \mathbf{d}_L) = p_L(I_L(\mathbf{p}, \mathbf{d}_L), N(\mathbf{p})) \quad (7)$$

3.1 Net photosynthetic rate

Net photosynthetic rate per unit leaf area (μmol $m^{-2}s^{-1}$) is characterised by a non-rectangular hyperbola [1]:

$$p_L(I,N)=\frac{\left(P_{\max}(N)+\phi I\right)-\sqrt{\left(P_{\max}(N)+\phi I\right)^2-4\theta P_{\max}(N)\phi I}}{2\theta}-R_d(N) \qquad (8)$$

where P_{max} is the maximum photosynthetic rate (μmol m^{-2} s^{-1}) and ϕ and θ are the quantum yield (mol mol^{-1}) and the curvature of the photosynthesis curve, respectively. P_{max} is assumed to be a curvilinear function of leaf N content:

$$P_{\max}(N)=\frac{\left(a_pN+b_p\right)c_p}{\left(a_pN+b_p\right)+c_p} \qquad (9a)$$

If this relation is assumed to be linear (depending on the measuring method) the asymptote is set to ∞, and eq.(9a) converges to:

$$P_{\max}(N)=a_pN+b_p \qquad (9b)$$

Dark respiration R_d (μmol $m^{-2}s^{-1}$) is given by:

$$R_d(N)=a_RN+b_R \qquad (10)$$

3.2 Nitrogen distribution

The leaf nitrogen content $N(\mathbf{p})$ (mmol m^{-2}) at any point **p** in the crown is characterised by an empirical relation between light attenuation and nitrogen distribution [2]:

$$N(\mathbf{p})=N_0\left(\frac{I(\mathbf{p})}{I_0}\right)^{a_N} \qquad (11)$$

where N_0 is the N content of a fully illuminated leaf in the top of the crown and a_N indicates the coefficient with which the nitrogen distribution scales with the light distribution. $I(\mathbf{p})$ is the photon flux density at point p, characterised as $I_n(\mathbf{p})/a_L$ (see next section) and I_o is $I(p)$ at the top of the crown ($\mathbf{p}$=0,0,c). Note that varying a_N-values result in a change in the total nitrogen content of the crown.

4 Photon absorption rate per unit volume

The photon absorption rate per unit volume I_n (μmol $m^{-3}s^{-1}$) is given by the integral:

$$I_n(\mathbf{p})=\int_{\Omega_L} f^{\Omega}(\mathbf{d}_L)I_L(\mathbf{p},\mathbf{d}_L)d\Omega_L \qquad (12a)$$

When rewriting this formula to spherical coordinates we get:

$$I_n(\mathbf{p})=\int_0^{\frac{\pi}{2}}\sin\vartheta_L f^{\Omega}(\vartheta_L)\int_0^{2\pi} I_L(\mathbf{p},\vartheta_L,\psi_L)d\psi_L d\vartheta_L \qquad (12b)$$

So the photon absorption rate per unit volume is determined by both the leaf area density and f^{Ω} in direction $\mathbf{d}_L$ and the net photon absorption rate I_L per unit leaf area (μmol $m^{-2}s^{-1}$) for leaves with leaf normal $\mathbf{d}_L$, which is:

$$I_L(\mathbf{p},\mathbf{d}_L) = a_L \int_{\Omega_i} \left| \langle \mathbf{d}_L, \mathbf{d}_i \rangle \right| i^{\Omega}(\mathbf{p},\mathbf{d}_i) d\Omega_i \qquad (13a)$$

or

$$I_L(\mathbf{p},\mathbf{d}_L) = a_L \int_0^{\frac{\pi}{2}} \sin(\vartheta_i) \int_0^{2\pi} \left| \langle \mathbf{d}_L(\vartheta_L,\psi_L), \mathbf{d}_i(\vartheta_i,\psi_i) \rangle \right| i^{\Omega}(\mathbf{p},\vartheta_i,\psi_i) d\psi_i d\vartheta_i \qquad (13b)$$

Here |<$\mathbf{d}_L$,$\mathbf{d}_i$>| is the magnitude of the inner product <$\mathbf{d}_L$,$\mathbf{d}_i$> of the leaf normal $\mathbf{d}_l$ and the unit length light direction vector $\mathbf{d}_i$. Symbol i^{Ω} denotes the light intensity per steradian coming from direction $\mathbf{d}_i$ and a_L is the leaf light absorption coefficient.

5 Photon flux density

To calculate the photosynthetic rate or the photon absorption rate for any point in the crown, the spherical light distribution must be specified. This directional photon flux density is determined by the extinction coefficient in that direction, the path lengths through the crown and the surrounding vegetation and the sky photon flux density for that direction.

5.1 Extinction coefficient and light attenuation

In general, the light attenuation through vegetation can be expressed as a differential equation of photon flux density per steradian from direction $\mathbf{d}_i$ over path length λ through the vegetation:

$$\frac{di^{\Omega}(\mathbf{d}_i)}{d\lambda} = -K(\mathbf{d}_i) f i^{\Omega}(\mathbf{d}_i) \qquad (14)$$

where the product of the light extinction K and the leaf area density f ($m^2\,m^{-3}$) is a function of the projection of all leaves in direction $\mathbf{d}_i$ multiplied by the leaf light absorption coefficient a_L:

$$K(\mathbf{d}_i) f = a_L \int_{\Omega_L} \left| \langle \mathbf{d}_L, \mathbf{d}_i \rangle \right| f^{\Omega}(\mathbf{d}_L) d\Omega_L \qquad (15a)$$

or

$$K(\mathbf{d}_i) f = a_L \int_0^{\frac{\pi}{2}} \sin(\vartheta_L) f^{\Omega}(\vartheta_L) \int_0^{2\pi} \left| \langle \mathbf{d}_L(\vartheta_L,\psi_L), \mathbf{d}_i(\vartheta_i,\psi_i) \rangle \right| d\psi_L d\vartheta_L \qquad (15b)$$

(Note that leaf area density per steradian f^{Ω} is independent of the azimuth angle.)
Solving eq.(14) results in a general expression for the directional light intensity:

$$i^{\Omega}(\mathbf{d}_i,\lambda) = i^{\Omega}_{fl}(\mathbf{d}_i) e^{-K(\mathbf{d}_i) f \lambda} \qquad (16)$$

where i^{Ω}_{fl} is the free sky light intensity per steradian (µmol $m^{-2}s^{-1}sr^{-1}$), f the leaf area density and λ the path length through the vegetation. Because the surrounding vegetation (index V) and the plant's crown (index E) may have different leaf area densities and leaf normal distributions, they may have different extinction coefficients. Eq.(16) should therefore be rewritten as:

$$i^{\Omega}(\mathbf{p},\mathbf{d}_i) = i^{\Omega}_{fl}(\vartheta_i)e^{-[K_E(\mathbf{d}_i)f_E\lambda_E(\mathbf{p})+K_V(\mathbf{d}_i)f_V\lambda_V(\mathbf{p})]} \tag{17}$$

5.2 Leaf area density

The leaf area density f (m^2m^{-3}) in terms of the leaf normal distribution at any point in the ellipsoid is given by the integral:

$$f = \int_{\Omega_L} f^{\Omega}(\mathbf{d}_L)d\Omega_L \tag{18}$$

To calculate the leaf area density distribution per steradian f^{Ω} (m^{-3}) from field data the total leaf area density f is split up and measured in a number of classes:

$$f = f_1 + f_2 + ... + f_n = f(f'_1 + f'_2 + ... + f'_n)$$

Here f'_k is the fraction of the total leaf area density with a normal in polar directions θ_k. For each direction class the density per steradian f^{Ω}_k is assumed to be uniform. Consequently, for each direction class k:

$$f^{\Omega}_k = \frac{f \cdot f'_k}{A_k} \tag{19}$$

with A_k the area on the unit sphere associated with the direction class k. The classes are defined by splitting the polar angle interval (0,2π) into intervals of equal width:

$$A_k = 2\pi(\cos\vartheta_{k-1} - \cos\vartheta_k) \tag{20}$$

with θ_k and θ_{k-1} the upper and lower bounds of interval k.

This division in classes makes f^{Ω} a non-continuous function. Consequently, in the numerical implementation the Gaussian integration over the azimuth angle ($d\theta$) of functions (15b) and (13b) is done separately over each leaf normal class.

6 Path lengths

The extinction of a light beam with direction $\mathbf{d}_i$ is determined by the path length of the beam through the crown and the surrounding canopy (Fig. 1).

6.1 Path length through the crown

Vector $\mathbf{p}=(p_x, p_y, p_z)$ denotes a vector to a point in the interior of the ellipsoid and vector $\mathbf{d}=(d_x, d_y, d_z)$ is a unit length vector specifying the direction for which the directional light intensity has to be calculated (Fig 1a). The length of the vector $\mathbf{p}$ to a point $\mathbf{q}$ on the boundary ∂E of the ellipsoid, pointing in direction $\mathbf{d}$, is written as $\lambda\mathbf{d}$. Since vector $\mathbf{d}$ has unit length, λ is the path length of the light beam through the tree crown. The directional path from $\mathbf{p}$ to point $\mathbf{q}$ can be parameterised by:

$$\mathbf{q}(\lambda) = \mathbf{p} + \lambda\mathbf{d} \tag{21}$$

with $\lambda \geq 0$. For the point of intersection $\mathbf{q}(\lambda)$ of this path with the boundary ∂E of the ellipsoid we get:

$$\frac{(q_x(\lambda))^2}{a^2} + \frac{(q_x(\lambda))^2}{b^2} + \frac{(q_x(\lambda))^2}{c^2} = 1 \qquad (22)$$

Expansion of this equation results in a quadratic expression for path length λ:

$$\alpha\lambda^2 + \beta\lambda + \gamma = 0 \qquad (23)$$

with:

$$\alpha = \frac{d_x^{\ 2}}{a^2} + \frac{d_y^{\ 2}}{b^2} + \frac{d_z^{\ 2}}{c^2}$$

$$\beta = \frac{2d_x p_x}{a^2} + \frac{2d_y p_y}{b^2} + \frac{2d_z p_z}{c^2}$$

$$\gamma = \frac{p_x^{\ 2}}{a^2} + \frac{p_y^{\ 2}}{b^2} + \frac{p_z^{\ 2}}{c^2} - 1$$

(Note that we are only interested in the positive root of this equation.)

6.2 Path length through the surrounding vegetation

Part of the path from **p** in direction **d** might be passing through the surrounding vegetation. Whether this is the case depends on the parameter values for the crown, gap radius and height of the surrounding vegetation. To illustrate the calculation of the path length of a light beam that passes through the vegetation, a sufficiently high vegetation is assumed with a gap having a radius that is larger than the semi-axes *a* and *b* of the crown. To calculate the path length in direction **d** for a point **p** on the boundary ∂E of the ellipsoid through the free space between the crown and the vegetation (Fig. 1b), the path is again described by an expression as given in eq.(21). For the point of intersection with the cylindrical gap side boundary we get:

$$(q_x(\lambda))^2 + (q_y(\lambda))^2 = r^2 \qquad (24)$$

Expansion results in a quadratic expression for λ:

$$\alpha\lambda^2 + \beta\lambda + \gamma = 0 \qquad (25)$$

with:

$$\alpha = d_x^{\ 2} + d_y^{\ 2}$$

$$\beta = 2p_x d_x + 2p_y d_y$$

$$\gamma = p_x^{\ 2} + p_y^{\ 2} - r^2$$

where λ is specified by the positive root of the equation.

The coordinate q_z determines whether the point of entry in the vegetation will be in the cylinder side boundary or in the vegetation bottom boundary. In this illustration, the point of intersection is in the cylinder side and consequently $h_b < q_z(\lambda) < h_t$.

To calculate the path length through the vegetation, a point **p** lying on the side boundary of the cylinder (Fig. 1c) is assumed. The length of the path from point **p** to point **q** on the upper boundary of the vegetation in direction **d** is specified by:

$$q_z(\lambda) = h_t \tag{26}$$

Substitution in eq. (21) results in the path length:

$$\lambda = \frac{h_t - p_z}{d_z} \tag{27}$$

7 Light distribution

Light intensity on top of the canopy derives directly from the way the light climate is defined.

External (free sky) light climate is given by:

$$i_{fl}^{\Omega}(\vartheta_i) = i_{fl,0}^{\Omega}(1-(\sin\vartheta_i)^c) \tag{28}$$

where i_{fl}^{Ω} is the free light intensity per steradian (μmol $m^{-2}s^{-1}sr^{-1}$) for direction $\mathbf{d}_i$, and $i_{fl,0}^{\Omega}$ is the light intensity per steradian coming from the zenith direction. The light intensity is a function of the polar angle of direction $\mathbf{d}_i$. This dependence on θ_i is given by the factor:

$$(1-(\sin\vartheta_i)^c)$$

in which c is an empirical parameter.

If c is sufficiently high, the light intensity i_{fl}^{Ω} will converge to the light intensity $i_{fl,0}^{\Omega}$ so that a uniform light intensity over the sky will be the result.

Light intensity per steradian $i_{fl,0}^{\Omega}$ from the zenith direction is related to the horizontal light intensity I_H (μmol $m^{-2}s^{-1}$) for the free sky by:

$$i_{fl,0}^{\Omega} = \frac{I_H}{2\pi\int_0^{\frac{\pi}{2}}\sin\vartheta_i\cos\vartheta_i(1-(\sin\vartheta_i)^c)d\vartheta_i} \tag{29}$$

For the analyses in this chapter empirical values I_H=1000 and c=2 are used.

8 Numerical integrations

All integrations described analytically above were implemented numerically in the form of Gauss-Legendre procedures [3]. For the analyses in this chapter the number of quadrature points for each integration was set to 6.

9 List of symbols

a, b, c	Semi-axes of crown ellipsoid *E* (m)
a_L	Leaf light absorption coefficient
a_N	Scaling coefficient of nitrogen distribution
a_p, b_p, c_p	Coefficients of the P_{max}–N relation
a_R, b_R	Coefficients of the R_d–N relation
A_k	Area on a unit sphere (m^2) for direction class *k*
d_i, ψ_i, θ_i	Cartesian light direction vector and its two spherical components
d_L, ψ_L, θ_L	Leaf normal vector and its two spherical components
E	Surface function of the crown ellipsoid
f	Leaf area density (m^2m^{-3})
f'_k	Fraction of the total leaf area density for direction class *k*
f^{Ω}	Leaf area density per steradian (m^{-3})
i^{Ω}	Light intensity per steradian (μmol $m^{-2}s^{-1}sr^{-1}$)
i_{fl}^{Ω}	Free light intensity per steradian (μmol $m^{-2}s^{-1}sr^{-1}$)
$i_{fl,0}^{\Omega}$	Free light intensity per steradian (μmol $m^{-2}s^{-1}sr^{-1}$) from zenith direction
I_0	Photon flux density at the top of the crown (μmol $m^{-2}s^{-1}$)
I_n	Light absorption rate per unit volume (μmol $m^{-3}s^{-1}$)
I_L	Light absorption speed per unit leaf area (μmol $m^{-2}s^{-1}$)
K_E, K_V	Extinction coefficients for crown and vegetation respectively
N	Nitrogen content of leaves (mmol m^{-2})
N_0	Nitrogen content of a fully illuminated leaf (mmol m^{-2})
P_{max}	Maximum (light saturated) photosynthetic rate (μmol $m^{-2}s^{-1}$)
P_E	Net photosynthetic rate of the crown (μmol s^{-1})
$P_L\, p_L$	Net photosynthetic rate per unit leaf area (μmol $m^{-2}s^{-1}$)
P_n	Net photosynthetic rate per unit crown volume (μmol $m^{-3}s^{-1}$)
R_d	Dark respiration (μmol $m^{-2}s^{-1}$)
λ_E, λ_V	Path length through crown and vegetation respectively (m)
ϕ, θ	Quantum yield and curvature of the photosynthesis curve

10 References

1. Johnson, I.R. and J.H.M. Thornley, *A model of instantaneous and daily canopy photosynthesis.* Journal of Theoretical Biology, 1984. **107**: p. 531-545.
2. Anten, N.P.R., *Modelling canopy photosynthesis using parameters determined from simple non-destructive measurements.* Ecological Research, 1997. **12**(1): p. 77-88.
3. Press, W.H., *Numerical recipes in Pascal.* 1989, Cambridge: University Press.

Figure 1 Three sets of vectors are used for path length calculations through crown (a), gap space (b) and surrounding vegetation (c).

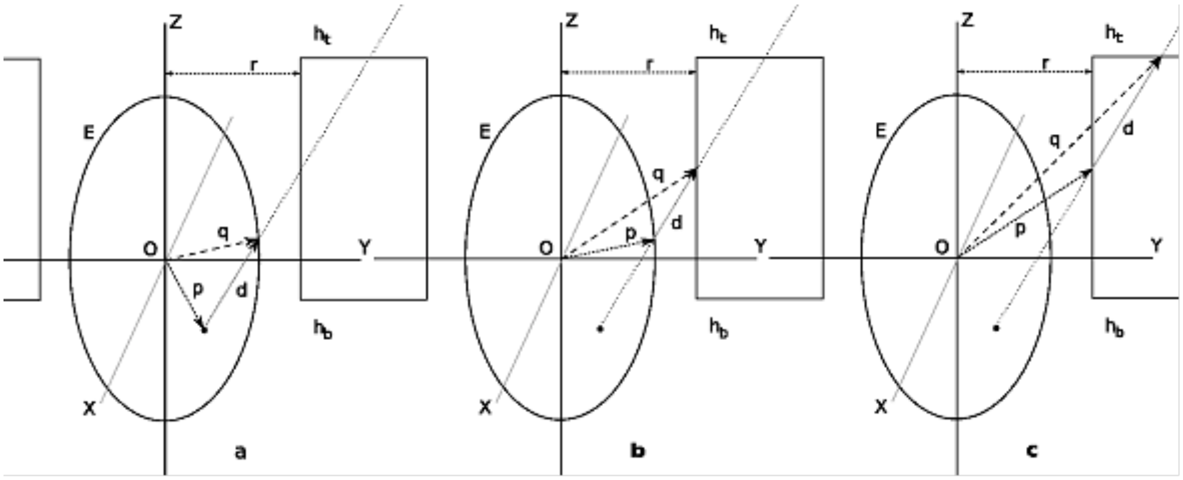

Figure 1
